# Functional Characterization of New Polyketide Synthase Genes Involved in Ochratoxin A Biosynthesis in *Aspergillus Ochraceus* fc-1

**DOI:** 10.3390/toxins7082723

**Published:** 2015-07-24

**Authors:** Liuqing Wang, Yan Wang, Qi Wang, Fei Liu, Jonathan Nimal Selvaraj, Lingna Liu, Fuguo Xing, Yueju Zhao, Lu Zhou, Yang Liu

**Affiliations:** 1 Institute of Food Science and Technology, Chinese Academy of Agricultural Sciences, 1 Nongda South Road, Xibeiwang Town, Haidian District, Beijing 100193, China; E-Mails: wanglq20082009@163.com (L.W.); wangyan062006@163.com (Y.W.); juwubawangqi@163.com (Q.W.); liufei0823@yeah.net (F.L.); sjonnim@gmail.com (J.N.S.); ling_na@yeah.net (L.L.); fgxing@163.com (F.X.); zhaoyueju@caas.cn (Y.Z.); zhoulu1982@sohu.com (L.Z.); 2 Key Laboratory of Agro-products Processing, Ministry of Agriculture, 1 Nongda South Road, Xibeiwang Town, Haidian District, Beijing 100193, China

**Keywords:** ochratoxin A, polyketide synthase, homologous recombination, biosynthetic pathway

## Abstract

Ochratoxin A (OTA), a potentially carcinogenic mycotoxin which contaminates grains, is produced by several *Aspergillus* species. A comparative sequence analysis of the OTA-producing *Aspergillus ochraceus* fc-1 strain and other *Aspergillus* species was performed. Two new OTA-related polyketide synthase (PKS) (*AoOTApks*) genes were identified. The predicted amino acid sequence of *AoOTApks-1* displayed high similarity to previously identified PKSs from OTA-producing *A. carbonarius* ITEM 5010 (67%; [PI] No. 173482) and *A. niger* CBS 513.88 (62%; XP_001397313). However, the predicted amino acid sequence of *AoOTApks-2* displayed lower homology with *A. niger* CBS 513.88 (38%) and *A. carbonarius* ITEM 5010 (28%). A phylogenetic analysis of the β-ketosynthase and acyl-transferase domains of the AoOTApks proteins indicated that they shared a common origin with other OTA-producing species, such as *A. carbonarius*, *A. niger*, and *A. westerdijkiae*. A real-time reverse-transcription PCR analysis showed that the expression of *AoOTApks-1 and -2* was positively correlated with the OTA concentration. The *pks* gene deleted mutants *∆AoOTApks-1* and *∆AoOTApks-2* produced nil and lesser OTA than the wild-type strain, respectively. Our study suggests that *AoOTApks-1* could be involved in OTA biosynthesis, while *AoOTApks-2* might be indirectly involved in OTA production.

## 1. Introduction

Ochratoxin A (OTA) is one of the most important mycotoxins produced by several species of *Aspergillus* and *Penicillium* that naturally occur in a variety of food commodities prior to harvest or, more commonly, during storage. OTA is a potent nephrotoxic mycotoxin [1], with the degree of renal injury observed depending on both the toxin dose and exposure time. OTA also displays hepatotoxic, teratogenic, and immunosuppressive activities [2,3,4]. Moreover it is categorized as a group 2B carcinogen by the WHO [5]. In many countries, regulatory limits regarding the presence of ochratoxin A in certain food commodities have been set. Among the ochratoxigenic fungi, *A. ochraceus* is considered one of the main fungi responsible for OTA contamination in several agricultural products. *A. ochraceus* contaminates many food commodities, including cereals, coffee, grapes, and others [6,7].

OTA consists of the amino acid phenylalanine linked by an amide bond to a pentaketide dihydroisocoumarin. However, recent results demonstrate that there are ambiguities in the OTA biosynthetic pathway [8]. Huff and Hamilton deduced the biosynthetic pathway based on the structure of OTA [9]. Nevertheless, Harris and Mantle demonstrated that mellein, which was proposed to be a OTA precursor by Huff and Hamilton [9], was not an intermediate and there was no evidence that ochratoxin C protected the phenylalanine carboxyl during OTA biosynthesis steps using ^14^C-labelled precursors and putative intermediates [10]. However, OTα, an intermediate probably recognized by Harris and Mantle [10], seemed to be a derivative of OTA [11]. Much less is known about the molecular genetic aspects of the OTA biosynthetic pathway. According to the OTA structure and its proposed biosynthetic pathway, OTA synthesis requires several proteins, including a polyketide synthase (PKS) for the biosynthesis of polyketide dihydroisocoumarin, a nonribosomal peptide synthetase (NRPS) for ligation of the amino acid phenylalanine and the polyketide, and a halogenase for chlorination. PKSs, as well as NRPSs, are large multimodular enzymes that play a role in the production of polyketide and peptide fungal secondary metabolites, respectively.

Complex and multifunctional PKSs are involved in the synthesis of most of fungal secondary metabolites. Despite a remarkable variety of final products, the individual polyketide biosynthetic reactions obviously follow a common basic principle. The key portion of these biosynthetic reactions is a repetitive decarboxylative condensation similar to the chain elongation step in fatty acid biosynthesis [12]. Based on their protein architectures, PKSs are classified into three basic types. Fungal PKSs are mainly termed iterative type I PKSs, and they have a modular organization like type I PKSs, but the catalytic reaction of each domain can act repeatedly. A typical PKS usually contains several conserved principal domains, β-ketoacyl synthase (KS), acyltransferase (AT), and acyl carrier protein (ACP), which catalyze the elongation of the polyketide chain. There are also some optional subunits, including β-ketoacyl reductase (KR), dehydratase (DH), enoyl reductase (ER), and thioesterase (TE), which are responsible for the production of polyketide products [13]. Based on the absence or presence of these reducing domain, PKSs in fungi are divided into non-reducing (NR) and highly reducing (HR) PKSs. Additionally, a third type of PKS, a partially reducing (PR) PKS, has been designated because of the absence of the ER domain.

In this study, based on the sequenced genome of *A. ochraceus*, two *pks* genes, designated *AoOTApks-1* and *AoOTApks-2*, were predicted to be involved in OTA biosynthesis. According to the alignment of OTA-PKS sequences and phylogenetic analyses of the KS and AT domains, the PKS encoded by *AoOTApks-1* was considered to be an HR PKS, while the PKS expressed by *AoOTApks-2* was classified as a member of the PR PKS, as it lacks a conserved ER domain. The expression profile of the two *AoOTApks* genes was consistent with OTA production during the growth of *A. ochraceus* fc-1. The deletion of *AoOTApks-1* via homologous recombination largely abolished OTA production. However, an analysis of the Δ*AoOTApks-2* mutant elucidated that the *pks-2* gene was possibly involved in OTA biosynthesis indirectly. Understanding the mechanistic differences between the two *pks* genes can help us to explore the OTA biosynthetic pathway and develop novel gene diagnostic strategies, based on *pks* genes, to prevent OTA contamination.

## 2. Results

### 2.1. Sequence and Phylogenetic Analysis of the Two AoOTApks Proteins

**Figure 1:**
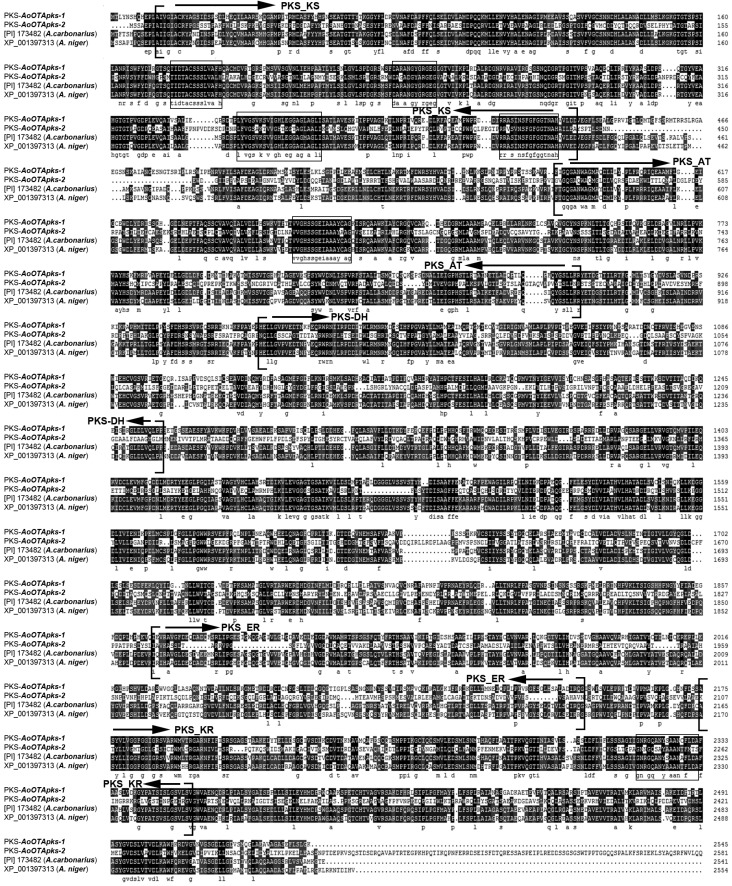
Alignment of the deduced amino acid sequences of OTA-PKSs in *A. ochraceus* fc-1 with homologous PKSs (protein identification [PI] no. 173482 and XP_001397313) in ochratoxin-producing *Aspergillus* species whose genomes have been sequence (*A. carbonarius* ITEM 5010 and *A. niger* CBS 513.88). Arrows indicate the conserved domains of PKSs according to the analyses of the Conserved Domain Database (CDD) and the Simple Modular Architecture Research Tool (SMART). Boxes indicate conserved regions of different functional domains.

The key *pks* genes associated with OTA biosynthesis in *A. ochraceus*, designated *AoOTApks*, were predicted by a comparative genomic analysis based on the genome-wide sequencing of *A. ochraceus* fc-1. Finally, two *pks* genes, designated *AoOTApks-1* and *AoOTApks-2*, which are probably involved in OTA biosynthesis, were identified. The deduced *AoOTApks* genes were predicted to display two open reading frames of 2617 (AoOTApks-1) and 3759 (AoOTApks-2) amino acids, and contains a typical conserved KS and AT domains (Figure 1 The predicted amino acid sequence of AoOTApks-1 displayed 67% identity to the PKS (protein identification [PI] No. 173482) from *A. carbonarius* ITEM 5010 and 62% identity to the PKS (XP_001397313) from *A. niger* CBS513.88. Meanwhile, the protein encoded by AoOTApks-2 showed 38% identity to the PKS (XP_001397313) from *A. niger* CBS513.88, and 28% identity to the PKS (protein identification [PI] No. 173482) from *A. carbonarius* ITEM 5010. AoOTApks-1 was 26% similar to AoOTApks-2 at the amino acid level.

**Figure 2:**
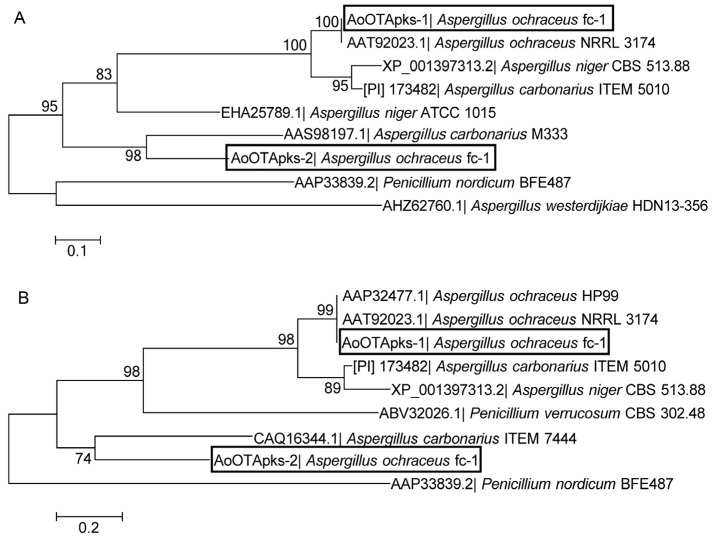
Maximum likelihood phylogenetic tree of (**A**) β-ketosynthase (KS) and (**B**) acyl-transferase (AT) domains from the deduced OTA-PKSs in ochratoxin-producing *Aspergillus* and *Penicillium* strains.

From the phylogenetic analyses of the key domains of the OTA-PKSs (Figure 2), the KS domain of AoOTApks-1 showed a higher similarity to KS domain in other PKSs from *A. carbonarius* and *A. niger*. Nevertheless, the KS domain of AoOTApks-2 displayed a lower identity with KS domains in other PKSs (AAS98197.1) from *A. carbonarius* M333. There was a similar phylogenetic tree outcome after analyzing the phylogeny and calibrating the AT domain of PKS. The AT domain of AoOTApks-2 showed a similarity with AT domains in other PKS (CAQ16344.1) from *A. carbonarius* ITEM7444. In the phylogenetic analyses of both domains, the OTA-PKS of *P. nordicum* BFE487 was relatively distant from most of the other PKSs identified in *Aspergillus* species. The PKS encoded by *AoOTApks-1* was similar to PKSs ([PI] no. 173482 and XP_001397313) that belong to the HR PKS clade, according to analyses of the protein by the Conserved Domain Database (CDD) [14] and Simple Modular Architecture Research Tool (SMART) [15]. However, the PKS expressed by *AoOTApks-2* was phylogenetically distant from the three aforementioned PKSs, because the protein belonged to one of the PR PKSs short of the conserved ER domain, just like the protein (AAP33839.2) in *P. nordicum* BFE487 [13]. The phylogenetic results confirmed both KS and AT domains (Figure 2).

### 2.2. Identification and Knockout of AoOTApks in A. Ochraceus

A deletion cassette for *AoOTApks* was designed, and the fusion PCR product, including the selective marker hygromycin phosphotransferase (*hph*) gene, was transformed into *A. ochraceus* fc-1 protoplasts.

**Figure 3:**
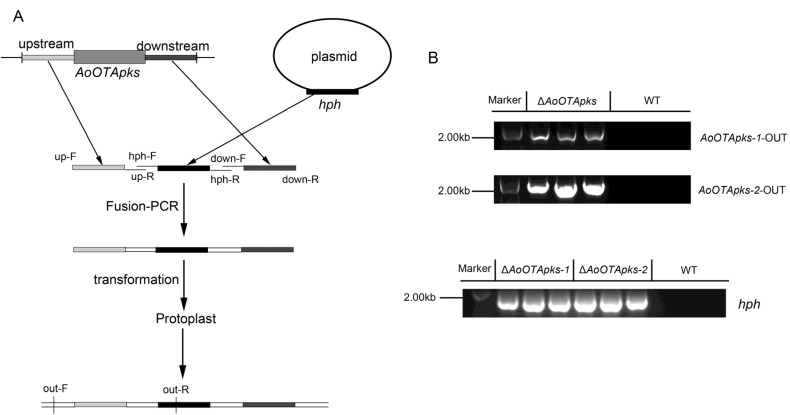
(**A**) Gene replacement strategy for the *pks* genes. Primer binding sites are indicated (see Table 1 and Table 2 for the primer sequences); (**B**) Confirmation of homologous recombination in the transformants was determined by PCRs using the *AoOTApks-1*-out and *AoOTApks-2*-out primers. The mutants were further confirmed using the *hph* primer pair.

Disruptions of *AoOTApks-1* and *-2* were separately confirmed by PCR analyses of the transformants (Figure 3B). The insertion of the selective marker was verified with the primer pair *hph*-F/R for both *pks* genes [16]. Additionally, deletions of *AoOTApks-1* and *-2* were separately confirmed with a primer pair (*AoOTApks-1*-out-F/R or *AoOTApks-2*-out-F/R; Table 1) designed to sequences upstream of *AoOTApks* and upto a partial *hph* gene fragment. All primers were designed using Primer Premier 6 (version 6.00, Palo Alto, CA, USA, America) and Primer-BLAST [17] software.

**Table 1:** Sequences of the primers used for DNA amplification in *A. ochraceus* fc-1.

Primer Name	Sequences (5ʹ to 3′)
*AoOTApks-1*-up-F	TCAGTAGACCAGTGAGGCGA
*AoOTApks-1*-up-R	CAAAATAGGCATTGATGTGTTGACCTCCCGGAGTTCGGGTGGTGATAG
*AoOTApks-1*-down-F	CTCGTCCGAGGGCAAAGGAATAGAGTAGAATCGCCCTCTCTTATGGCG
*AoOTApks-1*-down-R	GGGCTTGCTCAAAACTCTGC
*AoOTApks-1*-knock-F	GCTGGAATCGCGACAGAGTA
*AoOTApks-1*-knock-R	ACAGCCAAGCGCCAATTAGA
*AoOTApks-1*-out-F	ATGAGATACAGGAGCAAGC
*AoOTApks-1*-out-R	CACCAAGCAGCAGATGAT
*AoOTApks-2*-up-F	AATCCGTTCAGCTCCCCAAG
*AoOTApks-2*-up-R	CAAAATAGGCATTGATGTGTTGACCTCCGAAGGCATCGCCGTCAAATC
*AoOTApks-2*-down-F	CTCGTCCGAGGGCAAAGGAATAGAGTAGTACAGGTGCTACTTGCGTGG
*AoOTApks-2*-down-R	AGAAACGGTGTCCATCGTCC
*AoOTApks-2*-knock-F	GACAGATGGGATAGACGCCG
*AoOTApks-2*-knock-R	CTGAAAACGGGCAATTGGGG
*AoOTApks-2*-out-F	CACTCGGTCCTGCGTTAA
*AoOTApks-2*-out-R	GTCGTTCACTTACCTTGCTT
*hph*-F ^a^	GGAGGTCAACACATCAATGCCTATT
*hph*-R ^a^	CTACTCTATTCCTTTGCCCT

^a^ PCR primers for amplification of hygromycin resistance gene (*hph*) were referred according to the paper of Yun *et al.* [16].

**Table 2:** Sequences of the primers used for cDNA amplification in *A. ochraceus* fc-1.

Primer Name	Sequences (5′ to 3′)
*AoOTApks-1*-RT-F	CGCCTCATCATCAATCCTT
*AoOTApks-1*-RT-R	CAACTCGGTCAAGCAGAT
*AoOTApks-2*-RT-F	GGTGTGGCTGATGTAGTG
*AoOTApks-2*-RT-R	GTCTGTGAAGGTGTATGAATAG
GADPH-RT-F	GACTCACTATGCTGCCTAC
GADPH-RT-R	GAACCTTCTTGCCGTTGA

### 2.3. Ochratoxin A Production and Expression Analysis of AoOTApks in A. Ochraceus Grown on Corn Medium

**Figure 4:**
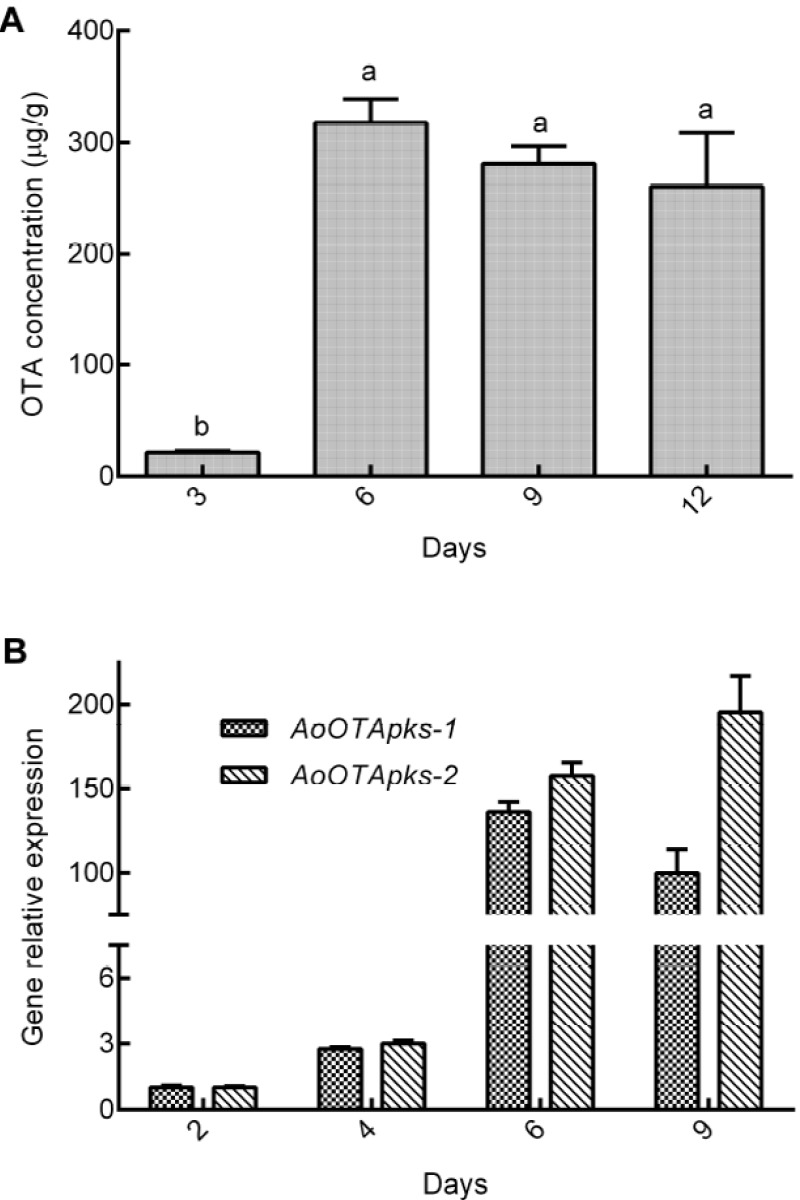
(**A**) The changes in the amount of OTA product at different time points during the growth of *A. ochraceus* fc-1. OTA concentrations were determined by HPLC-FLD using an OTA standard; (**B**) Relative expression of the *AoOTApks-1* and *-2* genes was assayed at different time points using a 7500 real-time PCR system. The glyceraldehyde 3-phosphate dehydrogenase (GADPH) gene was used as a control.

When *A. ochraceus* fc-1 was grown on corn, the OTA content, as analyzed by high-pressure liquid chromatography with a fluorescence detector (HPLC-FLD), was initially low, 21.30 μg/g of OTA was observed on the third day, and then sharply increased to 317.58 μg/g on the sixth day (*p* < 0.001; Figure 4). The OTA level was maintained higher between the sixth and twelfth day (*p* = 0.361).

The two *pks* genes were expressed at a relatively low level on the second day. However, their expression increased exponentially from the fourth to the sixth day. The highest expression level of *AoOTApks* was detected on the sixth day after inoculation, and it remained at the similar level between the sixth and ninth day (*p* = 0.041). The expression profile of the two putative *pks* genes at the mRNA level displayed a similar trend with OTA production during the growth of *A. ochraceus* fc-1.

### 2.4. Production of OTA in Mutant and Wild Type Strains of A. Ochraceus

**Figure 5:**
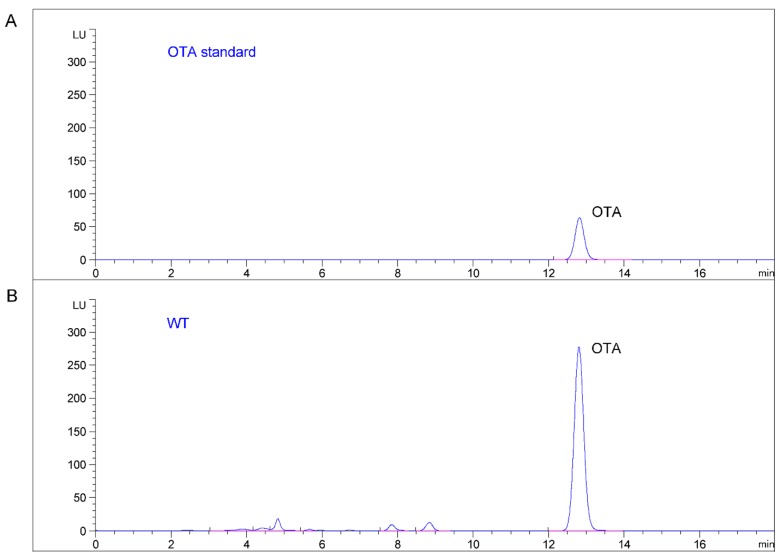

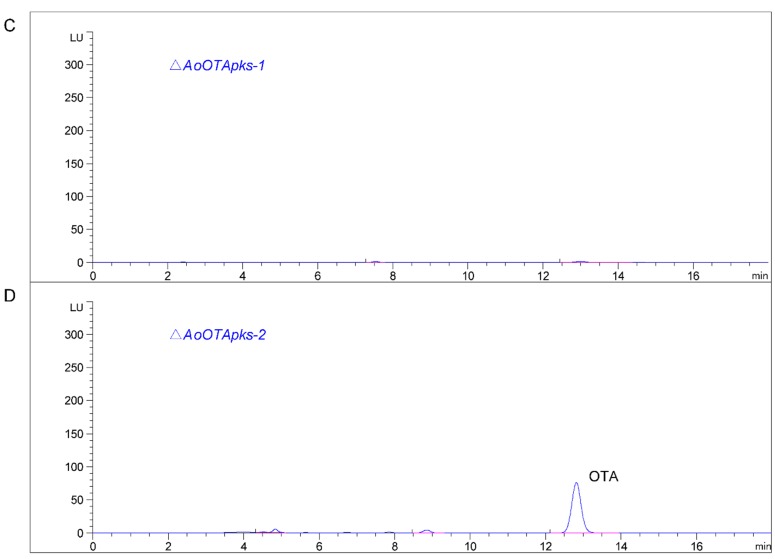
HPLC-FLD chromatograms of OTA. (**A**) OTA standard; (**B**) wild-type *A. ochraceus* fc-1; (**C**) the Δ*AoOTApks-1* mutant; (**D**) the Δ*AoOTApks-2* mutant. The y-axis of each profile is at the same order of magnitude.

**Figure 6:**
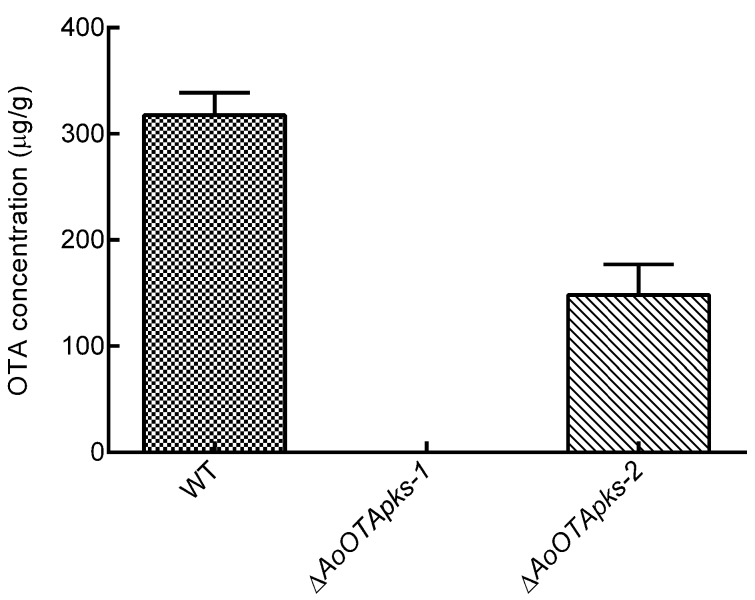
OTA production by wild-type (WT) *A. ochraceus* and the Δ*AoOTApks* mutants. The column separately means OTA production in wild type and the mutants inactivated *AoOTApks-1* or *AoOTApks-2* when the strains were respectively cultured on corn medium for 6 days.

Both *pks* deletion mutants and wild-type strains of *A. ochraceus* fc-1 were separately inoculated onto potato dextrose agar (PDA), and no differences in fungal growth, sporulation, or pigment production were observed between the wild-type and Δ*AoOTApks* strains (data not shown). To determine the OTA contents, the mutant and wild-type strains were also grown on corn medium. The OTA contents of both Δ*AoOTApks-1* and Δ*AoOTApks-2* were markedly decreased compared with that of the wild-type strain (Figure 5 and Figure 6). The mean value of OTA production in the wild-type culture was 317.58 μg/g, while the OTA contents of the Δ*AoOTApks-1* and Δ*AoOTApks-2* mutants were reduced by 100.0% (*p* < 0.001) and 53.8% (*p* = 0.004), respectively. The differences in the OTA production between the wild-type and mutant strains showed that both *pks* genes were probably involved in OTA biosynthesis.

## 3. Discussion

Genome-wide analyses of several filamentous fungi have been conducted recently [18], including several *Aspergillus* and *Penicillium* species, such as *A. flavus*, *A. nidulans*, *A. parasiticus*, *A. fumigatus*, *P. digitatum*, *P. expansum*, *P. nordicum*, *etc.* which produce several different mycotoxins. This information contributes to our understanding of their secondary metabolites. Among these filamentous fungi, the genomes of ochratoxigenic fungi, including *A. niger*, *A. carbonarius*, *A. parasiticus*, *P. nordicum*, and *P. verrucosum*, have been sequenced. The genomes of *A. carbonarius* ITEM5010 [19] and *A. niger* CBS513.88 [20] were analyzed in detail. Therefore, we conducted a comparative analysis of their genome sequences.

Most of the known mycotoxins produced by several fungi consist of a polyketide or peptide molecular structure catalyzed by PKS or NRPS [21]. Post-genomic analyses led to the identification of a number of PKSs, NRPSs, and hybrid PKS-NRPSs. These enzymes are involved in the biosynthesis of a large number of secondary metabolites, including mycotoxins. A chemical analysis of OTA, as well as its structure, indicated that five acetate units were incorporated into the dihydroisocoumarin moiety of OTA, starting from acetate and malonate, which is similar to the mechanism of fatty acid synthesis [12]. PKSs play an important role in the formation of the dihydroisocoumarin moiety. Mellein was initially suggested to be an intermediate of OTA biosynthesis [9]. Mellein is a metabolite that is also produced by ochratoxigenic species, and whose structure is similar to that of the dihydroisocoumarin portion of OTA, except the C7 carboxyl group. Despite this similarity, feeding experiments with ^14^C-labelled precursors did not support an intermediary role for mellein. Instead, the pentaketide 7-carboxymellein (OTβ) is supposed to be the most likely intermediate [10]. However, the nature of the PKS function in the OTA biosynthetic pathway has remained unsolved.

Based on the genomic sequence of *A. ochraceus* strain fc-1, *AoOTApks* genes were predicted and identified, in particular comparative analysis of putative *OTApks* genes from sequenced *A. niger* CBS513.88 and *A. carbonarius* ITEM5010. The two *OTApks* genes were not located in the vicinity. Gene disruptions revealed that the *AoOTApks* genes were required for OTA biosynthesis. *AoOTApks-1* probably performed the direct role in OTA production while *AoOTApks-2* maybe played a part in OTA biosynthesis indirectly.

The two PKSs encoded by the *AoOTApks* genes from *A. ochraceus* fc-1 both have typical, conserved KS and AT domains (Figure 1). They also have a DH domain and a KR domain according to the CDD [14] and the SMART [15]. The PKSs appear to have methyltransferase activity, which is likely required for the presence of the methyl group in the polyketide part of OTA. These conserved domains are also found in the OTA-PKSs in *A. carbonarius* ITEM5010 and *A. niger* CBS513.88. As the other OTA-PKSs are only partially sequenced to our knowledge, phylogenetic analyses of the KS and AT domains, which presumably play an important role in OTA biosynthesis, were carried out. Both PKSs in *A. ochraceus* fc-1 were shown to be responsible for mycotoxin biosynthesis. The KS and AT domains were required for OTA biosynthesis. According to the phylogenetic divergence of OTA-PKS conserved domains in *Penicillium* and *Aspergillus* species, a different evolutionary event could have occurred during the evolution of the *pks* genes involved in OTA biosynthesis.

The *pks* gene *aoks1* was confirmed to be required for OTA biosynthesis in *A. westerdijkiae* NRRL 3174, which was originally called *A. ochraceus* [22]. However, the predicted amino acid sequence of aoks1 displayed about 34% identity to OtapksPN in *P. nordicum* [23,24]. Later, the other PKS encoded by *aolc35-12* in *A. westerdijkiae* NRRL 3174 [22,25], was shown to control the expression of the *aoks1* gene required for OTA biosynthesis [22]. The PKSs encoded by *aolc35-12* and *aoks1* were approximately 34% homologous. It was deduced that *aolc35-12* could encode a certain polyketide compound which complements the expression of *aoks1* and, hence, the activation of the OTA biosynthesis system in *A. westerdijkiae*.

In *A. carbonarius* ITEM 7444, the *ACpks* gene was characterized, and the deduced protein was shown to contain conserved KS and AT domains [26]. There could be a correlation between the *ACpks* gene expression profile and OTA production, which suggests a likely role of PKSs in OTA biosynthesis in *A. ochraceus* fc-1 in our study. However, the other *pks* gene *AcOTApks* in *A. carbonarius* ITEM 5010, located next to the *AcOTAnrps* gene which is responsible for OTA biosynthesis [11], was deleted and the mutant had lost its ability to produce OTA [19]. Two *pks* genes related with OTA biosynthesis in *A. westerdijkiae* or *A. carbonarius* suggested that one *pks* gene was directly involved in OTA biosynthesis and the other *pks* regulated and complemented the expression of the former *pks* gene.

The expression of the two *AoOTApks* genes was monitored using qRT-PCR assays during the culturing and OTA production of *A. ochraceus* fc-1. Quantitative gene expression was used to understand the molecular functions of these genes, as well as the effects of biotic or abiotic factors on OTA production. In this study, the transcription of the two *pks* genes was monitored to analyze the potential relationship between the expression of the two *pks* genes and OTA production when *A. ochraceus* fc-1 was cultured on corn medium. An obvious positive correlation between the expression of the *AoOTApks* genes and OTA production was observed.

In a future study, the connection between *AoOTApks-1* and *AoOTApks-2* should be elucidated. There are also some instances of other fungal secondary metabolites whose biosynthesis requires two PKSs, such as zearalenone in *Fusarium* species [27,28], lovastatin in *A. terreus* [29,30], the T-toxin in *Cochliobolus heterostrophus* [31], compactin in *P. citrinum* [32], and asperfuranone in *A. nidulans* [33]. However, both *pks* genes may need to be disrupted or overexpressed to determine the potential connection between *AoOTApks-1* and *AoOTApks-2*.

In conclusion, two *pks* genes, designated as *AoOTApks-1* and *AoOTApks-2*, which were putatively involved in OTA biosynthesis in different manners, were described. The inactivation of *AoOTApks-1* and *AoOTApks-2* inhibited the OTA production. Analyses of gene expression and OTA production were also characterized during the growth of *A. ochraceus*. The connection between *AoOTApks-1* and *AoOTApks-2* will be studied in near future. The illustration of the key biosynthetic *pks* genes will not only contribute to explaining the pathway of OTA production, but will also provide necessary information for the development of effective gene diagnostic strategies to reduce the risk of OTA contamination in food and animal feed.

## 4. Experimental Section

### 4.1. Fungal Strains and Culture Conditions

*A. ochraceus* fc-1 is a high ochratoxin A-producing strain. This strain, as well as the two *pks* mutants (Δ*AoOTApks-1* and Δ*AoOTApks-2*) generated from it, was used throughout this study. To check the production of OTA and *pks* gene expression, 5 mL of conidia (10^6^ spores/mL) were inoculated into 100-mL Erlenmeyer flasks containing 25 g of corn.

### 4.2. DNA and RNA Extraction, cDNA Synthesis, qRT-PCR

For DNA extraction, *A. ochraceus* fc-1 was grown in 50 mL of yeast extract-sucrose (YES) liquid medium at 28 °C on a horizontal shaker (180 rpm). After 4–7 days of incubation, mycelia were collected and dried. Then, the mycelia were ground in liquid nitrogen using the Fungal DNA kit (E.Z.N.A., Omega Bio-Tek, Norcross, GA, USA) according to the manufacturer’s instructions.

The cultured samples were ground with a pre-cooled mortar and pestle according to a previously published method [34], with some modifications. One hundred mg of the powder was treated and mixed with 1.5 mL of TRIzol (Life Technologies, Carlsbad, CA, USA). Total RNA extraction was performed using TRIzol according to the Cold Spring Harbor protocol [35].

qRT-PCR was conducted in a 7500 Real-Time PCR system (Applied Biosystems, Foster City, CA, USA). Reactions were prepared in triplicate in MicroAmp optical 96-well reaction plates, and sealed with optical adhesive covers (Applied Biosystems, Foster City, CA, USA). Three replicates of control sample without cDNA were also included in the runs. The SYBR green I protocol was performed in a final volume of 20 μL, containing 10 μL of Power SYBR-Green PCR Master Mix, 2 μL of cDNA template, 0.6 μL of the primer pairs (*AoOTApks-1*-RT-F/R, *AoOTApks-2*-RT-F/R, and GADPH-RT-F/R, 10 μmol/L each; Table 2), and 7.4 μL of sterile deionized water.

Real-time PKS gene expression was monitored using a 7500 Real Time PCR system (Applied Biosystems). It was programmed to hold at 95 °C for 5 min, and to complete 40 cycles of 95 °C for 25 s, 55 °C for 35 s, and 72 °C for 35 s. For real-time PCRs with SYBR Green I, a melting curve stage was programmed to check the expected amplification products. The thermal protocol for dissociation was defined as 15 s at 95 °C, 1 min at 60 °C, 1% increments of the slow ramp rate between 60 and 95 °C, 30 s at 95 °C and 15 s at 60 °C after the real-time PCR cycles.

The expression levels of the different mRNAs were evaluated by comparing their Ct values. The relative quantification of gene expression was established using the comparative 2^−ΔΔCT^ method.

### 4.3. Amino Acid Alignments and Phylogenetic Analyses of OTA PKSs

To identify the KS and AT domains, the amino acid sequences of the putatively analogous PKSs were analyzed with the CDD [14] and the SMART [15]. The amino acid sequences of the KS and AT domains were aligned with the ClustalW algorithm using MEGA 6 software [36]. Genealogy of the KS and AT domains on the basis of the obtained alignment was inferred by a maximum likelihood analysis using MEGA 6. A phylogeny test was run using the bootstrap method with 1,000 replications.

### 4.4. Disruption of AoOTApks Genes and Construction of the ∆AoOTApks Mutants

To construct the *AoOTApks-1* and *-2* deletion strains, 1.3–1.5 kb of upstream and downstream DNA fragments from the promoter and terminator regions were separately cloned and designated as flanking sequences (Figure 3A). The *hph* cassette was used in the binary vector pCAMBIA-1300 as a selective marker. The *hph* gene was cloned using the primer pair *hph*-F/R. The two amplified flanking sequences and the *hph* gene cassette were mixed and amplified by fusion PCR using knockout primers (*AoOTApks-1*-knock-F/R, *AoOTApks-2*-knock-F/R; Table 1). The fusion PCR products were respectively transformed into *A. ochraceus* fc-1 protoplasts, from which the cell wall had been removed by enzymatic digestion with snailase, cellulase, and lysozyme, using via a polyethylene glycol (PEG)-mediated transformation. The cell wall was regenerated in regeneration medium (27.4% sucrose, 0.1% casein hydrolysate, and 0.1% yeast extract), and then the strain was transferred to PDA plates containing hygromycin B (80 μg/mL) as the selective agent for fungal transformants.

Disruption of *AoOTApks-1* and *-2* was confirmed by PCR analyses of the transformants (Figure 3B). The insertion of the selective marker was checked with the primer pairs *AoOTApks-1*-RT-F/R, *AoOTApks-2*-RT-F/R and also *hph*-F/R (Table 1 and Table 2).

### 4.5. Detection and Measurement of OTA by HPLC-FLD

To determine the OTA concentration of the corn medium on which the three *A. ochraceus* fc-1 strains (wild type, Δ*AoOTApks-1*, and Δ*AoOTApks-2*) were grown, cultures were ground and 5 g of the powder samples was separately collected. Then, the samples were resolved in 25 mL of methanol, mixed well and filtered. Ten mL of the supernatant was evaporated with nitrogen gas at 60 °C using a pressure blowing concentrator. The metabolites were resolved in 2 mL of methanol and filtered through a 0.22-μm filter. The filtrate was finally employed to test the OTA concentration using HPLC-FLD. The final OTA concentration of the cultured corn medium was calculated using the following formula:

OTA final concentration (μg/g) = OTA determination concentration (μg/mL) × 2 mL/10 mL × 25 mL/5 g.

OTA concentrations were assayed, and 20 μL of each sample was injected into the HPLC-FLD apparatus (Agilent Technologies, Santa Clara, CA, USA). The HPLC-FLD apparatus was a 1260 infinity (LC) system comprising a binary pump, an autosampler, and a fluorescence detector (excitation wavelength, 333 nm; emission wavelength, 460 nm [37]). The column was an Agilent TC-C18(2) column (250 mm × 4.6 mm, 5-μm particles) (Agilent, Santa Clara, CA, USA). The mobile phase was a mixture of acetonitrile, water, and acetic acid (99:99:2). The flow rate of the mobile phase was 1 mL/min. For the sample determinations, an OTA standard curve was determined by detecting 0.1, 1.0, and 10.0 μg/mL of OTA standard.

### 4.6. Statistical Analysis

All the statistics were analyzed by SPSS Statistics 21.0 (version 21.0, IBM, Armonk, NY, USA). The OTA contents and *pks* gene expression analyses were evaluated using one-way analysis of variance (ANOVA). Mean differences were determined by Tukey’s post-hoc tests (*p* = 0.01). All the figures were plotted by GraphPad Prism 6 (version 6.02, La Jolla, CA, USA).
